# Christian Science(?)

**Published:** 1892-06

**Authors:** 


### Selections.

### CHRISTIAN SCIENCE (?)

Scarcely a week passes in which there is not recorded the death of some patient of the so-called "Christian Scientists." One of the latest cases comes from the Pacific coast. According to the *Occidental Medical Times* great interest has been excited in the neighborhood of San Bernardino, Cal., over the trial of Mrs. Eliza Ward, a "Christian Science Healer," on the charge of manslaughter for having caused the death of George Lord, Jr., by gross neglect and want of ordinary caution and circumspection.

The facts of the case appear to be about as follows: Lord was attacked by a violent headache, superinduced by the accumulation of pus in the frontal sinuses and ethmoidel cells, the pus finding its way into the orbital cavity, forcing the eye outward and downward about half an inch from its normal position. The pus also passed upwards; forcing a sac above the left eye, two and a half inches in length by one inch in depth. Meningitis supervened, resulting in death. Mrs. Ward had considerable influence with the wife of the victim, and persuaded her that if she would exclude all the physicians she could bring "her husband out all right." This was done, and at the end of five days the patient died, notwithstanding that Mrs. Ward all this time insisted that he would soon be at work again.

In the prosecution the following facts were brought to light: Mrs. Davis, a Christian Science expert, testified that "science" treated strychnine poisoning, the bite of a rattle-snake, small-pox, cholera, and fevers all alike. She denied that actual disease could be cured, but said that people only had "a belief in disease." She admitted that surgeons were needed in cases of broken bones, but said that in the case of the deceased surgery was not admitted by "science," and that the half or two-thirds of a cup of pus that had accummulated would, by "science," be allowed to remain in his system.

Cincinnati has had several such deaths, and there is a prospect of many others in the future. The narration of cases like the above brings into prominence the fact that the laws of the different States regarding medical practice are extremely defective. While restrictions have been imposed in Illinois and other States upon the irregular doctor of the old-fashioned type, no effective legal barriers have been erected against the advent of the new order of "Scientists," who are thus allowed full liberty to experiment with human life and to populate cemeteries. Any fool or crank can herald himself a "Christian Scientist" and start forth to care for the lives of men. There is no present method of limiting the number of these "scientists," nor of imposing a medical education upon them. It is a rather remarkable fact that in this, the greatest protection country of the world, free trade, in dealing with human life and the practice of medicine, reigns as in no other country. We protect ourselves against merchandise, but not against persons.

The intelligence of the age demands statutes prohibiting men and women from engaging in any form of medical practice without first acquiring a thorough knowledge of the various branches of medicine. Our experience with the Ohio Legislature this last winter has convinced us that it is a waste of time and words and money to talk about protecting the people against anything. The people think that they know how to protect themselves. The public seems imbued with the idea that the efforts of physicians to secure legislation demanding a thorough medical education is but a selfish effort. The press, also, has always been found battling against that which would legislate away its gains.

In regard to the San Bernardino case mentioned above, it may be interesting to note that the jury, after remaining out all night, finally returned a verdict of not guilty, notwithstanding that the case was skilfully prosecuted, and the elements of involuntary manslaughter were clearly proven, and the court charged the jury, if they found that the life of deceased had been shortened by reason of the gross carelessness ot the defendant, that she was guilty, even though they might further find that when in the right mind deceased consented to place his life in the care of the defendant. The general sentiment was that the defendant ought to have been punished; but she was a woman, and many ladies had stood by her.—*Lancet-Clinic.*

